# Maintenance treatment with interferon for advanced ovarian cancer: results of the Northern and Yorkshire gynaecology group randomised phase III study

**DOI:** 10.1038/sj.bjc.6602037

**Published:** 2004-08-10

**Authors:** G D Hall, J M Brown, R E Coleman, M Stead, K S Metcalf, K R Peel, C Poole, M Crawford, B Hancock, P J Selby, T J Perren

**Affiliations:** 1 Cancer Research UK Clinical Centre in Leeds, St James's University Hospital, Leeds, UK; 2 Clinical Trials and Research Unit, Leeds University, Leeds, UK; 3 Academic Unit of Clinical Oncology, Weston Park Hospital, Sheffield, UK; 4 Princess Anne Hospital, Southampton, UK; 5 Department of Gynaecological Oncology, Leeds Teaching Hospitals NHS Trust, Leeds, UK; 6 City Hospital, Dudley Road, Birmingham, UK; 7 Airedale General Hospital, Skipton Road, Steeton, Keighley, UK

**Keywords:** ovarian cancer, interferon-alpha, maintenance therapy

## Abstract

A randomised phase III trial was conducted to assess the role of interferon-alpha (INF*α*) 2a as maintenance therapy following surgery and/or chemotherapy in patients with epithelial ovarian carcinoma. Patients were randomised following initial surgery/chemotherapy to interferon-alpha 2a as 4.5 mega-units subcutaneously 3 days per week or to no further treatment. A total of 300 patients were randomised within the study between February 1990 and July 1997. No benefit for interferon maintenance was seen in terms of either overall or clinical event-free survival. We conclude that INF-*α* is not effective as a maintenance therapy in the management of women with ovarian cancer. The need for novel therapeutics or strategies to prevent the almost inevitable relapse of patients despite increasingly effective surgery and chemotherapy remains.

Although epithelial ovarian cancer is a relatively chemosensitive disease, the disease ultimately relapses and leads to the death of most patients. The median duration of remission following first-line chemotherapy with platinum/taxane combinations for patients with advanced disease is between 12 and 24 months. A variety of strategies have been employed to maintain the benefits achieved with chemotherapy in advanced ovarian cancer. The dose and duration of chemotherapy have both been increased, but increases in response rates have not been associated with improvement in survival (Eltabbakh *et al*, 1998; Umesaki *et al*, 1999; McGuire, 2000).

The concept of additional therapy to maintain the response achieved with chemotherapy has been assessed in a number of disease and clinical settings. In the 1980s, interferon-alpha (INF*α*) was assessed as a maintenance therapy for patients with multiple myeloma. As a single agent, interferon has a low response rate in myeloma patients with responses of approximately 15%. However, in patients who demonstrated no evidence of disease progression following primary chemotherapy, maintenance therapy with low-dose subcutaneous INF*α* improved progression-free and overall survival (Mandelli *et al*, 1990).

Interferon-*α* has limited activity in active advanced ovarian cancer (Freedman *et al*, 1983; Einhorn *et al*, 1988). However, we hypothesised that subcutaneous INF*α* may act as an effective maintenance therapy in patients with advanced ovarian cancer and thus improve overall survival. To test this hypothesis, a prospective randomised trial was performed in patients with FIGO stage Ic–IV epithelial ovarian cancer, in whom no evidence of disease progression was seen following surgery and/or chemotherapy.

## PATIENTS AND METHODS

**Figure 1:**
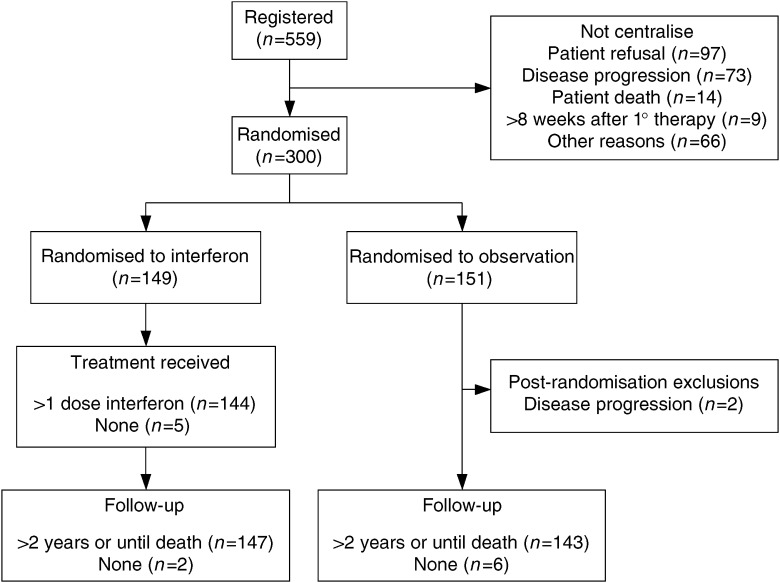
Trial profile.

The study was approved by the Local Research Ethics Committee of each participating centre and written informed consent was obtained prior to randomisation. Patients eligible for the study had histologically proven epithelial ovarian cancer that showed no evidence of disease progression (on clinical, radiological or CA-125 assessment) following postoperative chemotherapy. Patients were randomised within 8 weeks of completing chemotherapy to treatment with INF-*α* 2a (Roferon-A, Roche) (4.5 mega-units subcutaneously 3 days per week) or to a no maintenance treatment control group. Interferon was continued until disease progression, or in response to toxicity or patient request. The study was designed to detect a 50% increase in median survival and therefore required 300 randomised patients. The trial profile is shown in Figure 1

.

To further analyse the effect of maintenance interferon, the disease status of each patient was determined following surgery and/or chemotherapy. Patients in whom there was no evidence of disease either clinically or radiologically following surgery and/or chemotherapy were designated as disease-free (DF). Those patients in whom disease was apparent clinically or radiologically following surgery and/or chemotherapy were designated as disease-present (DP).

For additional detail regarding patient selection, dose modifications, study design and statistical methods, see supplementary information.

## RESULTS

Between February 1990 and July 1997, 300 patients were randomised by 23 consultants from 14 centres across the UK (for participating centres and consultants, see supplementary information), 151 to observation, 149 to interferon. The patients in the two treatment arms were similar with respect to baseline characteristics (Table 1

**Table 1:** Patient characteristics by treatment group

	**Interferon**	**Observation**
	**(*n*=149)**	**(*n*=149)**
*Median (range) age at diagnosis (years)*	58 (31–76)	57 (33–78)
		
*FIGO stage at diagnosis*		
I	11 (7%)	12 (8%)
II	22 (14%)	19 (13%)
III	94 (63%)	95 (64%)
IV	22 (15%)	23 (15%)
		
*Residual disease following surgery (cm)*		
0	23 (18%)	26 (20%)
<2	44 (35%)	51 (40%)
2–5	24 (19%)	13 (10%)
>5	36 (28%)	40 (31%)
		
*Histology*		
Serous	67 (45%)	72 (48%)
Mucinous	10 (7%)	12 (8%)
Endometrioid	27 (18%)	34 (23%)
Clear cell	4 (3%)	4 (3%)
Other	20 (13%)	10 (7%)
Undifferentiated	3 (2%)	2 (1%)
Not stated	18 (12%)	15 (10%)
		
*Tumour grade*		
1	11 (9%)	14 (11%)
2	47 (38%)	43 (33%)
3	66 (53%)	75 (57%)
Not stated	25	17
		
*WHO performance status pre-chemotherapy*		
0	84 (60%)	83 (56%)
1	46 (32%)	46 (31%)
2	7 (4%)	17 (12%)
3	3 (2%)	1 (1%)
4	3 (2%)	0
Not stated	6	2
		
*Chemotherapy regimen*		
Platinum single agent	89 (60%)	88 (59%)
Platinum combination	44 (29%)	37 (25%)
Platinum/taxane	15 (10%)	20 (13%)
Taxane single agent	0	1 (1%)
Other single agent	1 (1%)	3 (2%)
		
*Response to surgery and/or chemotherapy*		
Disease free	89 (61%)	95 (65%)
Disease present	58 (39%)	52 (35%)
Not evaluable	2	2
		
*CA125 after surgery and/or chemotherapy*		
<35 U ml^−1^	47 (77%)	62 (78%)
35–65 U ml^−1^	2 (3%)	9 (11%)
65–200 U ml^−1^	8 (13%)	5 (6%)
>200	4 (7%)	4 (5%)
Not stated	88	69
		
*WHO performance status at randomisation*		
0	113 (76%)	114 (79%)
1	32 (22%)	28 (19%)
2	3 (2%)	2 (1%)
3	0	0
4	0	0
Not stated	1	5

). The median follow-up time for interferon patients was 27.0 months (range 2.3–149.7), and for observation patients 32.2 months (range 1.7–141.7). Eight patients (two interferon and six observation) died without attending any follow-up visits. Of the 149 patients randomised to receive maintenance INF*α*, 144 received at least one injection (see supplementary information). The duration of treatment and the reason for stopping is shown in Table 2

**Table 2:** Duration of interferon treatment and reason for stopping treatment

	**Number of patients**	**Median (range) time (in months) on interferon**
*All patients*	144	5 (<1–88)
		
*Interferon stopped*		
Disease progression	61	8 (1–66)
Toxicity	50	3 (<1–37)
Patient desire	14	7 (<1–48)
Other	8	38 (1–71)
Death of patient	4	8 (3–14)
Not stated	4	22 (7–51)
*Treatment status unknown at last review*	3	54 (51–88)

. Figure 4 shows compliance with interferon maintenance over time in those patients who remained progression free. The most commonly reported toxicity/adverse event for both interferon and observation patients was fatigue (72 and 46%, respectively) (Table 3

**Table 3:** Adverse events/toxicity (all grades)

	**Interferon**	**Observation**	
	**(*n*=144)**	**(*n*=149)**	**Significance**
Toxicity/adverse event			
Flu-like symptoms	102 (69%)	47 (31%)	*P*<0.001
Fatigue	106 (71%)	71 (47%)	*P*<0.001
Nausea/vomiting	61 (41%)	52 (34%)	Not significant
			
Neurological	6 (4%)	NR	—
Dyspnoea	9 (6%)	NR	—
Depression/anxiety	9 (6%)	NR	—
Skin change/rash	7 (5%)	NR	—
Alopecia	7 (5%)	NR	—
Arthalgia/arthritis	4 (3%)	NR	—
Insomnia	3 (2%)	NR	—
Haematological	3 (2%)	NR	—
Hepatic	1 (1%)	NR	—
Injection site reaction	2 (1%)	NR	—
Other	19 (13%)	NR	—
Not stated	4 (3%)	NR	—

NR=not recorded.

). Dose modification of interferon was made in 18 (12.5%) patients due to toxicity.

**Figure 2:**
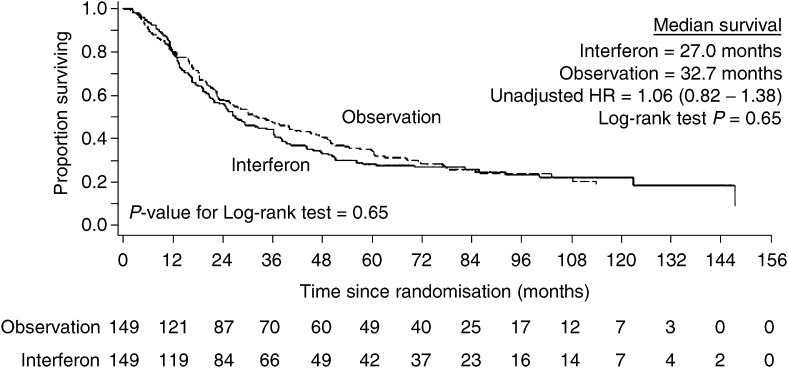
Overall survival by randomised treatment group.

No benefit for interferon maintenance was seen in terms of either overall or clinical event-free survival. The median overall survival in the interferon arm was 27.0 months and in the observation arm 32.7 months. The median clinical event-free survival in the interferon arm was 10.3 months and 10.4 months in the observation arm. The Kaplan–Meier survival curves for overall and clinical event-free survival (Figure 2

and Figure 3

**Figure 3:**
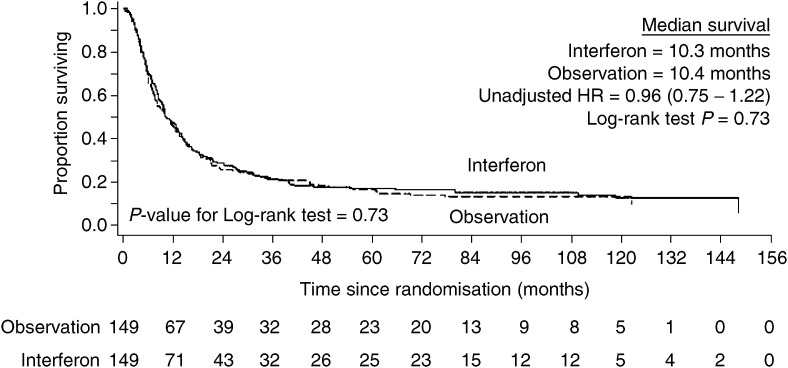
Clinical event-free survival by randomised treatment group.

) showed no significant difference (overall survival – unadjusted HR=1.06, 95% C.I.=0.82–1.38, Log-rank test *P*=0.65; clinical event-free survival – unadjusted HR=0.96, 95% C.I.= 0.75–1.22, Log-rank test *P*=0.73). When hazard ratios were adjusted for prognostic factors previously defined, the results were similar.

The effect of treatment on overall survival and clinical event-free survival was compared separately among patients who were DF after surgery and/or chemotherapy and those who had DP after surgery and/or chemotherapy. This comparison was unplanned and performed only as a hypothesis generating analysis. No significant differences for interferon maintenance were identified (DF patients – clinical event-free survival – unadjusted HR=0.88, 95% C.I.=0.63–1.22).

The multivariate analysis of prognostic factors and treatment received, performed on the 298 patients in the intention to treat population (Table 4

**Table 4:** Multivariate analysis of treatment received and prognostic factors for overall and clinical event-free survival (*n*=298)

	**Overall survival**		**Clinical event-free survival**	
	**Adjusted HR (95% C.I.)**	***P***	**Adjusted HR (95% C.I.)**	***P***
*Randomised treatment*				
Observation	1.00		1.00	
Interferon	1.10 (0.83–1.45)	0.51	0.92 (0.71–1.20)	0.55
				
*Response–1° treatment*				
Disease-free	1.00		1.00	
Disease-present	1.53 (1.12–1.65)	0.01	1.84 (1.37–2.49)	<0.001
				
*Age at diagnosis (years)*				
<55	1.00		1.00	
55–65	1.51 (1.10–2.07)		1.13 (0.83–1.52)	
>65	1.36 (0.92–2.01)	0.034	1.14 (0.79–1.65)	0.67
				
*FIGO stage*				
Ic	1.00		1.00	
II	0.80 (0.33–2.74)		1.02 (0.51–2.06)	
III	2.11 (1.04–4.29)		2.48 (1.28–4.80)	
IV	2.82 (1.32–6.03)	<0.001	2.92 (1.42–5.59)	<0.001
				
*WHO performance status*				
0	1.00		1.00	
1	1.16 (0.82–1.65)		1.16 (0.82–1.65)	
2/3/4	1.74 (1.22–2.49)	0.035	1.74 (1.22–2.49)	0.62
				
*Size of residual disease*				
0	1		1	
<2 cm	1.02 (0.63–1.64)		1.02 (0.63–1.64)	
>2 cm	1.10 (0.67–2.44)		1.09 (0.67–1.77)	
Not stated	1.69 (0.97–2.93)	0.17	1.69 (0.97–2.93)	0.74
				
*Tumour type*				
Serous	1.00		1.00	
Mucinous	1.32 (0.78–2.22)		1.15 (0.68–1.93)	
Endometrioid	1.16 (0.79–1.70)		1.06 (0.72–1.56)	
Clear cell	1.69 (0.65–4.41)		1.39 (0.49–3.93)	
Other	0.86 (0.53–1.40)		0.89 (0.56–1.43)	
Undifferentiated	1.09 (0.37–3.81)		1.36 (0.52–3.57)	
Not stated	1.59 (1.00–2.43)	0.33	1.39 (0.87–2.21)	0.74
				
*Tumour grade*				
Well differentiated	1.00		1.00	
Moderately differentiated	0.83 (0.45–1.54)		0.87 (0.54–1.52)	
Poorly differentiated	1.33 (0.73–2.41)		1.32 (0.77–2.24)	
Not stated	0.92 (0.46–1.84)	0.033	0.85 (0.45–1.60)	0.038

), demonstrated that receiving interferon maintenance was not related to either overall or clinical event-free survival (see supplementary information).

## DISCUSSION

This trial was designed to determine whether the use of low-dose subcutaneous INF*α* could improve the overall survival of patients with epithelial ovarian cancer following primary therapy with surgery and/or chemotherapy. Patients with all stages of disease at presentation and with no evidence of disease progression after primary therapy were randomised within the trial. The overall and clinical event-free survival of ovarian cancer patients studied within this trial was typical of results obtained with platinum combination chemotherapy in the 1990s. However, survival times were not improved by the addition of maintenance low-dose subcutaneous IFN*α* following primary therapy.

Interferon-*α* has been shown to have an *in vitro* activity against ovarian cancer cell lines (Epstein *et al*, 1980) and a limited clinical benefit in advanced ovarian cancer (Freedman *et al*, 1983; Einhorn *et al*, 1988). Clinical studies have assessed the effect of intraperitoneal INF*α* in a number of phase II studies both after and in combination with platinum chemotherapy (Berek *et al*, 1985; Nardi *et al*, 1990; Willemse *et al*, 1990; Bruzzone *et al*, 1997; Berek *et al*, 1999). These studies have confirmed that such a treatment can be delivered although its benefit over standard treatment has not been assessed. No clinical trial of subcutaneous INF*α* in ovarian cancer has previously been reported. However, an alternative immunoregulatory cytokine, interferon-*γ* has been assessed in a randomised phase III trial as an addition to primary chemotherapy with cisplatin and cylophosphamide (Windbichler *et al*, 2000). This showed a significant improvement in progression-free survival. However, improvements in clinical response rate and overall survival did not achieve statistical significance.

Experience with biological therapy has suggested that the benefits of such therapy are often seen in patients with small volume or clinically undetectable disease. Although this effect has also been seen in previous trials of cytokine therapy in ovarian cancer (Berek *et al*, 1985; Pujade-Lauraine *et al*, 1996), the recent study of interferon-*γ* showed equal efficacy in patients with both small and large volume disease (Windbichler *et al*, 2000). To mirror the initial study of interferon maintenance in myeloma, patients with no evidence of disease progression following primary therapy were randomised and therefore, many patients with residual disease were randomised. However, a subgroup analysis of the patients in complete clinical remission within this study failed to identify a significant benefit for those patients.

**Figure 4:**
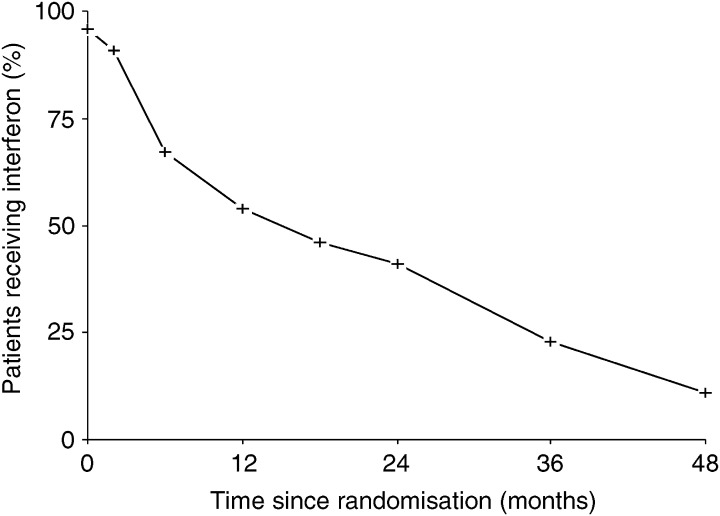
Compliance with interferon maintenance. The percentage of patients still receiving interferon in the absence of disease progression is shown.

Compliance with interferon maintenance was poor (Figure 4

). At 6 months, only 67% of patients with no evidence of disease progression remained on interferon maintenance. This is likely to be due to the fact that this cohort of patients had undergone both surgical intervention and chemotherapy prior to commencing interferon treatment. This is in contrast to studies in myeloma, renal cancer and melanoma in which treatment rarely follows both extensive surgery and chemotherapy. Pegylated interferon has recently been introduced into clinical trials. The half-life of these modified forms of interferon are significantly increased allowing weekly administration. It may be that the use of such treatment would improve compliance by reducing both the frequency of administration and the toxicity associated with the treatment.

Despite modern chemotherapy combinations, the natural history of ovarian carcinoma remains one of relapse following initial response to chemotherapy. A potential benefit for maintenance paclitaxel chemotherapy for some patients with ovarian cancer has recently been reported although at the cost of clinically significant toxicity (Markman *et al*, 2003). The need for an effective nontoxic maintenance therapy is clear. This study has demonstrated that subcutaneous INF-*α* given following surgery and chemotherapy is not the answer to this ongoing dilemma. However, the recent development of new, targeted molecular therapies may allow the concept of maintenance therapy to be examined further.
