# Supplementary material for: Maintenance treatment with interferon for advanced ovarian cancer: results of the Northern and Yorkshire gynaecology group randomised phase III study
Source: Br J Cancer. 2004 Aug 10;91(4):621–6. doi: 10.1038/sj.bjc.6602037 (PMC2364769; doi:10.1038/sj.bjc.6602037)
Supplement: Supplementary Information [file 91-6602037x1.doc]

## Supplementary information - Patients and methods

### Patient selection

Patients were initially eligible for registration with the trials office following the diagnosis of histologically proven epithelial ovarian cancer (FIGO stages III and IV) that required post-operative chemotherapy. A protocol modification in 1993 extended the study to include patients with FIGO stages Ic and II disease who had been treated with adjuvant chemotherapy. The extent of surgery and choice of chemotherapy was not defined within the protocol although maximal surgical debulking followed by chemotherapy was recommended. The 1993 protocol modification advised a platinum-based regimen. It was the intent of the study co-ordinators that patients potentially suitable for the study should be registered with the clinical trials office. However, registration at the time of diagnosis was not mandatory or required for subsequent randomisation within the study, indeed many patients were randomised without prior registration. Patients were randomised within the trial if, following surgery and/or chemotherapy, there was no evidence of disease progression i.e. patients had achieved a complete response, partial response or stable disease. This was assessed clinically, radiologically and by sequential CA125 measurement. Patients were required to have had no previous treatment other than surgery and standard chemotherapy. Registered patients in whom progressive disease was diagnosed were not randomised within the trial and were subsequently treated at their clinician’s discretion. The study was approved by the Local Research Ethics Committee of each participating centre. Written informed consent was obtained prior to randomisation.

### Doe modifications

Interferon dose was reduced by 25% in response to WHO Grade 2 toxicity at the discretion of the investigator. In all patients with WHO Grade 3 or 4 toxicity interferon was immediately stopped until resolution of symptoms. Interferon was then resumed at no more than 50% of the previous dose. Although the duration of interferon was not directly specified within the protocol, the information sheet advised that treatment should be for a minimum of one year and for longer in patients who were tolerating treatment well.

### Study design

The trial was a multi-centre, non-blinded randomised study. Randomisation was stratified by centre and by chemotherapy trial, if applicable, using random permuted blocks. Patients were randomised via a central telephone system administered by the Clinical Trials and Research Unit (CTRU) ensuring a concealed, secure system.

Surgery and chemotherapy were performed according to the normal, local ovarian cancer treatment policy. No central review of pathology or response was required in this study.

A subsequent study was commenced in 1995 assessing Quality of Life using the Rotterdam Symptom Checklist, the Hospital Anxiety and Depression Scale and the Sexual Activity Questionnaire ( de Haes *et al*, 1990; Zigmond *et al*, 1983; Thirlaway *et al*, 1996).

Following randomisation, patients were monitored for adverse events and disease status every two months for the first year, every three months for the second year, every four months for the third year and every six months for subsequent years. In addition to clinical assessment, abdominal imaging (CT or ultrasound) was performed every six months for the first two years or on clinical suspicion of relapse. All adverse events were recorded for patients receiving interferon. Only the frequency of flu-like symptoms, fatigue and nausea/vomiting was recorded for patients on the observation arm of the trial. Progression was defined clinically, radiologically or by an increase in CA125 (of more than two-fold with at least two measurements taken at the higher level). Radiological confirmation of clinical or CA125 evidence of disease progression was sought where possible.

Overall survival was defined as the time from randomisation to death from any cause. Clinical-event free survival was defined as the time from randomisation to either the first appearance of progressive disease detected clinically (i.e. diagnosed by clinical examination, CA125 or imaging) or to death from any cause (which ever came first). Patients still alive at the time of analysis were censored at the date of last follow-up; patients lost to follow-up were censored at the date of last follow-up. The trial profile is shown in Figure 1.

### Statistical Methods

The median survival, based on available information at the time, for this group of patients was expected to be 12 to 18 months, with only 10% of patients alive at 5 years. 300 patients were recruited to detect an improvement in 5 year survival of approximately 10% (or an improvement of 6-9 months in median survival) at a 5% significance level with 85% power. The date of analysis was September 2003.

For the main treatment comparisons, the Kaplan-Meier method was used to estimate survival curves, and survival times were compared using the log-rank test. Cox’s proportional hazards regression was used to estimate hazard ratios adjusted for pre-defined prognostic factors (response to treatment, age at diagnosis, FIGO stage at diagnosis, post-chemotherapy WHO performance status, size of residual disease, tumour type, and tumour grade). In all analyses, a hazard ratio (HR) greater than 1 implied patients treated with interferon were at greater risk of progressing/dying relative to observation patients. Toxic events for patients who took one or more doses of interferon were summarised and compared to patients in the observation arm. The chi-square test was used to test the difference in proportions for flu-like symptoms, fatigue and nausea/vomiting.

To further analyse the effect of maintenance interferon, the disease status of each patient was determined following surgery and/or chemotherapy. Patients in whom there was no evidence of disease either clinically or radiologically following surgery and/or chemotherapy were designated as disease-free (DF). Those patients in whom disease was apparent clinically or radiologically following surgery and/or chemotherapy were designated as disease-present (DP).

All analyses were done by intention to treat, except in the analyses of toxic events. All p-values were two-sided and all analyses were performed using SAS version 8 (SAS UK).

de Haes JC, van Knippenberg FC & Neijt JP (1990) Measuring psychological and physical distress in cancer patients: structure and application of the Rotterdam Symptom Checklist. *Br J Cancer* **62:** 1034-1038.

Thirlaway K, Fallowfield L & Cuzick J (1996) The Sexual Activity Questionnaire: a measure of women's sexual functioning. *Qual Life Res* **5:** 81-90.

Zigmond AS & Snaith RP (1983) The hospital anxiety and depression scale. *Acta Psychiatr Scand* **67:** 361-370.

## Supplementary information - Participating centres and consultants

Birmingham - Queen Elizabeth Medical Centre (C Poole). Bradford - Bradford Royal Infirmary (C Bradley, D Parker). Bristol - Bristol Haematology and Oncology Centre (J Graham). Huddersfield - Huddersfield Royal Infirmary (J Campbell, J Joffe). Hull - Princess Royal Hospital (C Preston). Keighley - Airdale General Hospital (SM Crawford). Leeds - Cookridge Hospital (S Cartwright, H Close, RI Rothwell), Leeds General Infirmary (EJ Buxton, M Glass, K Hancock, N Johnson, K Peel), St James’s University Hospital (G Lane, T Perren). Merseyside - Clatterbridge Centre for Oncology (J Green). Middlesbrough - James Cook University Hospital (A Rathmell). Portsmouth - St Mary’s Hospital (G Khoury). Scarborough - Scarborough Hospital (M Noble, D Poole). Sheffield - Northern General Hospital (M Paterson), Weston Park Hospital (R Coleman). Northallerton - Friarage Hospital (F Bryce, M Kumarendran). Wakefield - Pinderfields General Hospital (M Shields).

## Supplementary information - Compliance

149 patients were randomised to receive maintenance INFα. Five (3%) of these patients did not receive interferon; two patients demonstrated disease progression between randomisation and the date they were due to start interferon, one refused interferon after randomisation, another withdrew consent 3 days after randomisation, the reason for the fifth is unknown.

## Supplementary information - Prognostic factor multi-variate analysis

### Prognostic factors

A multivariate analysis of prognostic factors and treatment received was performed on the 298 patients in the intention to treat population (Table 4). Disease presence after surgery and/or chemotherapy was related to both an increased risk of death (p=0.008) and an increased risk of experiencing a clinical event (p<0.001). An increased FIGO stage was also related to both an increased risk of death (p<0.001) and an increased risk of experiencing a clinical event (p<0.001). Increased age at diagnosis (p=0.034) and an increased WHO performance status (p=0.035) were also related to an increased risk of death. The treatment to which a patient was randomised was not related to either overall or clinical event-free survival. The multivariate analysis was repeated on a sub-group of patients for whom CA125 data was available (n=137). In this sub-set of patients, the baseline CA125, i.e. the level at the time of randomisation was shown to be the most significant independent variable for overall and clinical event-free survival (p<0.001).
